# Production, optimization, scale up and characterization of polyhydoxyalkanoates copolymers utilizing dairy processing waste

**DOI:** 10.1038/s41598-024-52098-0

**Published:** 2024-01-18

**Authors:** Tejaswini Dhanaji Patil, Saptaneel Ghosh, Aparna Agarwal, Sanjay Kumar Singh Patel, Abhishek Dutt Tripathi, Dipendra Kumar Mahato, Pradeep Kumar, Petr Slama, Ales Pavlik, Shafiul Haque

**Affiliations:** 1 Department of Dairy Science and Food Technology, Institute of Agricultural Sciences, Banaras Hindu University, Varanasi, Uttar Pradesh 221005 India; 2 Department of Food and Nutrition Science, Lady Irwin College, Delhi University, New Delhi, 110001 India; 3 Department of Chemical Engineering, Konkuk University, Seoul, 05029 Republic of Korea; 4 School of Exercise and Nutrition Sciences, CASS Food Research Centre, Deakin University, Burwood, VIC 3125 Australia; 5 Department of Botany, University of Lucknow, Lucknow, 226007 India; 6 Department of Animal Morphology, Physiology and Genetics, Faculty of AgriSciences, Mendel University in Brno, 61300 Brno, Czech Republic; 7 Research and Scientific Studies Unit, College of Nursing and Health Sciences, Jazan University, Jazan, 45142 Saudi Arabia; 8 Gilbert and Rose-Marie Chagoury School of Medicine, Lebanese American University, Beirut-1102 2801, Lebanon; 9 Centre of Medical and Bio-Allied Health Sciences Research, Ajman University, Ajman-13306, United Arab Emirates

**Keywords:** Biotechnology, Chemical biology, Environmental sciences

## Abstract

The microbial biotransformation using low-cost feedstock to produce biopolymers (degradable), an alternative to petrochemical-based synthesis plastics (non-degradable), can be a beneficial approach towards sustainable development. In this study, the dairy industry processes waste (whey) is used in polyhydroxyalkanoate (PHA) copolymer production. Initial screening suggested that *Ralstonia eutropha* produced higher PHA as compared to *Bacillus megaterium*. A central composite rotatable design-based optimization using two process variables (amino acid and tween-80) concentration remarkably influenced PHA co-polymer production under physiological conditions of pH (7), temperature (37 °C), and agitation rate of 150 rpm. High polyhydroxybutyrate (PHB) mass fraction yield of 69.3% was observed as compared to predicted yield of 62.8% from deproteinized whey as feed. The combination of tryptophan (50 mg L^−1^) and tween-80 (3 mL^−1^) enhanced *R. eutropha* mass gain to 6.80 g L^−1^ with PHB contents of 4.71 g L^−1^. Further, characterization of PHA and its copolymers was done by ESI–MS, FTIR, and TEM. On upscaling up to 3.0 L, the PHA contents and yields were noted as quite similar by *R. eutropha*. This study demonstrates that dairy waste processing waste can be potentially utilized as inexpensive feed for producing high content of biopolymers to develop a sustainable system of waste management.

## Introduction

There is a considerable need for biodegradable plastic nowadays to save nature. So, our lives as conventional non-biodegradable plastic are causing severe environmental threats. Production of biodegradable plastics is increasing daily, which is being used in different sectors like agriculture, pharmaceutical, cosmetics, and food industries. Therefore, researchers are focusing more on producing biodegradable plastics like PHA, which can be degraded by the microbial populations present in the soil or the environment^1^. The rapidly rising demand for biobased packaging locates PHA to bring down carbon footprints and save Mother Earth by reducing plastic pollution. 100% biodegradable, water-insoluble PHAs are obtained from prokaryotic microorganisms as an intracellular reserve material synthesized under nutrient-limiting conditions except for carbon which is the primary substrate for this PHA^2^. PHA polymer, produced by three different pathways in microorganisms as storage molecules, is present in different forms, like stiff, brittle, and rubbery, depending upon their chemical composition and these pathways requires three different precursors like acetyl CoA, malonyl CoA, acyl-ACP which are synthesized from sugar, oils and fatty acids, respectively (Proenca et al.; 2023). Unlike polylactic acid (PLA), PHA is UV stable^3^. Various kinds of microbial species can produce these inclusions in the cell’s cytoplasm. Bacteria can accumulate up to 90% of PHB on a cellular dry-weight basis in their cytoplasm^4^. Bacterial such as *Bacillus*, *Ralstonia* (formerly known as *Cupriavidus*, *Wautersia,* or *Alcaligenes*), *Pseudomonas*, *Burkholderia*, *Comamonas*, *Thermus*, and *Escherichia* are used in the production of PHA^5^. Due to PHA's attention-seeking properties resembling petroleum polymers except for biodegradability, it can be used in food packaging, medical, pharmaceutical, or other biotechnological applications^6^.

Right now high production cost of PHAs like P3HB (poly-3-hydroxybutyrate), PHBV (poly-3-hydroxybutyrate-co-3-hydroxy valerate), P3HP (poly-3-hydroxypropionate), P3HHx (poly-3-hydroxyhexanoate), P4HB (poly-4-hydroxybutyrate), and P5HV (poly-5-hydroxy valerate) compared to conventional polymers like polypropylene, polyethylene, and polyethylene terephthalate is the primary limiting for its broader application in the packaging industry. Therefore, PHAs can be produced either by using waste material as an organic substrate or by developing new fermentation, recovery, purification, and characterization techniques or by using such strains which are capable of biosynthesis of a high quantity of PHAs^7,8^. Previous studies have revealed that PHA can be produced by utilizing carbon-rich sources using suitable microorganisms from waste materials of agriculture-based industries, sugar manufacturing industries such as molasses^3^, oil refineries, marine industries, dairy, and food industries which produce colossal waste. Dairy industry waste is increasing in parallel with the increase in milk production and dairy product plants and therefore, their waste management is also an important area of interest^9^. Lower production costs can be achieved by using dairy industry waste which provides a huge amount of sweet whey rich in lactose and whey proteins. Although, microbes don't require nitrogen in a higher amount for PHA production as biosynthesis occurs in nitrogen-limiting conditions, is one of the above-mentioned alternative ways, but it results in lower yield than pure organic substrate^10^.

Various strategies have been demonstrated to improve biopolymer accumulation via screening of PHA-producers, optimization of process parameters (carbon/nitrogen source, pH, temperature, time, and agitation rate), and utilization of sugars as feed. Using inexpensive feed, such as biowaste, an alternative to costly pure sugars, can be helpful in enhancing PHA properties and bioprocess economy. In the present study, the PHA-producing potential of *Bacillus megaterium* and *Ralstonia eutropha* were evaluated from dairy processing waste under diverse physiological conditions. Furthermore, variables such as amino acid and tween-80 concentrations were optimized to enhance the PHA co-polymers production by *R. eutropha* as an efficient PHA-producers utilizing whey supernatant as carbon and nitrogen source under submerged fermentation.

## Materials and methods

### Cultures and growth conditions

PHA-producing cultures *Bacillus megaterium* (MTCC 428) and *Ralstonia eutropha* (MTCC 8320) were procured from Microbial Type Culture Collection, (MTCC) Chandigarh, India. These cultures were grown in a complex medium [peptone (10 g L^−1^), beef extract (10 g L^−1^), and ammonium chloride (5 g L^−1^)], and maintained on agar (2 0 g L^−1^) plates by every month sub-culturing.

### Pretreatment of whey for feed preparation

The whey acquired during the manufacturing of paneer was pretreated through acidification to remove excess proteins prior to utilization as a carbon source (whey lactose), and the procedure details are illustrated in Supplementary Figure S1^11^. The whey supernatant pH was adjusted to 7 (1 N NaOH/HCl) and used as feed for bacterial growth and PHA production.

### Submerged culture PHA accumulation

PHA production by *B. megaterium* and *R. eutropha* was performed in 250 mL Erlenmeyer flasks with 100 mL of medium containing deproteinized whey (pH, 7) which was replaced with fructose (40, g L^−1^) for the control growth media. The production medium was prepared by adding mineral salt medium consisting of urea (0.8, g L^−1^), KH_2_PO_4_ (2.0 g L^−1^), Na_2_HPO_4_ (0.6, g L^−1^), MgSO_4_.7H_2_O (1.0, g L^−1^), yeast extract (0.1 g L^−1^), and trace element solution [1 mL/L of ZnSO_4_.7H_2_O (1.3, g L^−1^), CaCl_2_ (20, g L^−1^), FeSO_4_.7H_2_O (0.2, g L^−1^), (NH_4_)_6_Mo_7_O_24_.4H_2_O (0.6, g L^−1^), and H_3_BO_3_ (0.6, g L^−1^)], and deproteinized whey (pH, 7). Fully grown culture (5%, v/v) was inoculated to medium and incubated for up to 72 h for PHA accumulation at 37 °C under an agitation rate of 150 rpm. Further, the influence of carbon source, and process parameters (pH, temperature, and the agitation rate on PHA accumulation were evaluated for incubation of 48 h.

### Supplementation of amino acids

To improve the growth and PHA accumulation, the supplementation of various amino acids [aromatic amino acids (tyrosine, tryptophan, and phenylalanine) and sulfur-containing amino acids (cystine, cysteine, and methionine)] were assessed at a concentration of 1 mg/1 mL of deproteinized whey. The resulting feed was inoculated with cultures (5%, v/v) to measure their influence on biomass and PHA production for incubation of 48 h at 37 °C under an agitation rate of 150 rpm.

### Effect of different pH of production medium on growth and PHB production

In all experiments, pH was maintained at 7 but to check the effect of pH on the biomass and PHB yield pH of the production medium is adjusted to pH 5, 7, 9, and 11 using 1N NaOH/1N HCl. The inoculum was prepared as described previously. After preparation of the media, 5 ml of inoculums were inoculated in 100 ml of production medium. The flasks were incubated at 150 rpm at 37 °C. At 24 h, 48 h, and 72 h, biomass and PHB were measured in the culture broth.

### Effect of different temperatures of fermentation on growth and PHB production

Production medium inoculated with two different strains in two separate 250 ml flasks. The flasks were incubated at 150 rpm at different temperature ranges such as 25 °C, 30 °C, 33 °C, 37 °C, and 40 °C. Biomass and PHB were determined in the culture broth at 24 h, 48 h, and 72 h.

### Effect of different agitation speeds during fermentation on growth and PHB production

Here, the production medium made up of whey was inoculated with two different bacterial strains in two separate flasks. Then the flasks were incubated at 37 °C at different ranges of agitation speeds such as 50 rpm, 100 rpm, 150 rpm, and 200 rpm. Biomass and PHB were measured in the culture broth at different time intervals such as 24 h, 48 h, and 72 h.

### Effect of different times of fermentation on growth and PHB production

To check the effect of time of fermentation on biomass growth and PHB production, a previously prepared production medium containing whey was inoculated with 5 ml bacterial inoculums of different strains in a 250 ml flask separately and the cell culture was incubated at 150 rpm at 37 °C. Biomass and PHB were estimated in the culture broth at different time intervals such as 48 h, 60 h, and 72 h. The production medium in each flask contained whey only.

### Experimental design and validation

To determine the interaction impact of two physical process factors (amino acid and tween-80) concentration on PHA accumulation, a two-factor central composite rotary design (CCRD) was employed using software (Design Expert 12.0, Stat-Ease Inc., USA). At various levels of two parameters, 13 sets of experiments were created by design experts (Table 1). Point prediction was used to adjust each factor's level for maximum performance. Experimental testing was done to determine the model’s effectiveness using the combination of several optimal parameters that generated the highest reaction, or maximum PHA content.

**Table 1:** Design of experiment for cell mass and PHA production using stat-ease software.

Run	Factor 1	Factor 2	Response 1	Response 2
	Tryptophan (mg/L)	Tween 80 (%, v/v)	Cell mass (g/L)	PHA yield (g/L)
1	37.5	4.5	6.11 ± 0.21	4.10 ± 0.16
2	25.0	6.0	5.83 ± 0.17	4.09 ± 0.14
3	37.5	6.6	6.33 ± 0.23	4.90 ± 0.17
4	37.5	4.5	6.09 ± 0.20	4.07 ± 0.11
5	37.5	4.5	6.24 ± 0.24	4.02 ± 0.13
6	37.5	4.5	6.20 ± 0.18	4.00 ± 0.14
7	37.5	4.5	6.42 ± 0.27	4.05 ± 0.10
8	50.0	3.0	6.81 ± 0.23	4.71 ± 0.12
9	37.5	2.4	6.06 ± 0.16	3.97 ± 0.09
10	25.0	3.0	5.43 ± 0.12	3.82 ± 0.13
11	50.0	6.0	6.74 ± 0.24	4.78 ± 0.19
12	55.2	4.5	6.47 ± 0.25	4.54 ± 0.15
13	19.8	4.5	5.41 ± 0.17	3.56 ± 0.16

### Analytical measurements

DCW content of the biomass was measured by cell drying procedures to achieve a constant weight at 80 °C for incubation of 24 h^12^. Whey protein and total sugar contents were measured by procedures of Lowery^13^, and 3,5-dintrosalicylic acid (DNSA)^14^, respectively. PHA accumulation was assessed using sodium hypochlorite (50 mL) and chloroform (50 mL) dispersion procedure from retrieved cells (1.0 g dry cell weight)^15^. The obtained crude PHA was precipitated in a non-solvent solution [70% methanol, v/v] and recovered via filtration (Whatman No. 1 paper) followed by drying for incubation of 5 h at 70°C^15^. PHA contents of cultures were measured by chloroform extraction followed by spectrophotometrically at 235 nm estimation of crotonic acid as described earlier^16^.

### PHA characterization

The Fourier transform infrared (FTIR) spectra of PHA granules were recorded by Bruker spectrometer (Thermo Nicolet, MA, USA). Electrospray ionisation mass spectrometry (ESI–MS) analysis was evaluated by a Finnigan LCQ ion trap mass spectrometer (Thermo Finnigan LCQ Fleet, San Jose, CA, USA)^17^. The intracellular PHA granules of the cells were recorded by transmission electron microscopy (TEM) using glutaraldehyde (2%) procedures as described earlier^18^.

### Up-scaling of PHA production

The culture was grown in a 7.5-L Bentchtop bioreactor (BioFlo/Celligen 115, New Brunswick, USA) to study the up-scaling of PHA production with a working volume of 3-L under optimized conditions.

## Results and discussion

### PHA production from whey hydrolysate

The whey composition analysis reveals that it contains approximately 6.8% of total sugars (lactose, glucose, and galactose) and traces of essential growth factors (calcium and phosphorus) Pure sugars have been widely reported as a feed for PHA production. Therefore, low-cost biowaste materials such as whey can improve process economics^19^. In this study, *B. megaterium* and *R. eutropha* as PHA-produces were employed to produce PHA under submerged culture from whey hydrolysate as a carbon source. The PHA production details of these cultures from various carbon sources are presented in Table 2. The cell biomass of *B. megaterium* and *R. eutropha* improved to 4.54 and 6.05 g/L by using whey hydrolysate, which is greater than 3.55 and 3.94 g/L that was obtained after using pure sugars like glucose and fructose as carbon sources. These carbon sources resulted in PHA yields of up to 2.93 g/L for *B. megaterium* and 3.84 g/L for *R. eutropha*. Here, the PHA contents in cell biomass noted entirely consist in the range of 61.4–65.3% for *B. megaterium* and 61.1–63.5% for *R. eutropha*. Previously, a PHA production of 1.1 g/L by *Paracoccus homiensis* from cheese whey mother liquor (CWML)^20^, 3.32 g/L by *Bacillus mycoides* DFC1 from glucose^21^, and 4.01 g/L by *Alcaligenes latus* (ATCC 29714) from sugar beet juice supplemented with minerals^22^, 1.69 g/L by *Bacillus firmus* NII 0830 from acid pretreated rice straw hydrolysate^23^, and 2.7 g/L by *Hydrogenophaga pseudoflava* from whey lactose^24^ were obtained. Growth factors (organic acids, vitamins, and minerals) present in whey positively supported cell biomass and PHA production. Higher cell mass and increased PHA productivity clearly indicate that these PHA-producers assimilated nitrogen from whey efficiently and the uptake rate across cell membrane may be higher due to its non-ionic form and less pH dependency during transport across the membrane. Based on the high production of cell biomass (6.05 g/L) and PHA (3.84 g/L) by *R. eutropha* over *B. megaterium*, *R. eutropha* was selected for further studies.

**Table 2:** PHA production potential of *B. megaterium* and *R. eutropha* from various cabon sources.

Carbon source	*B. megaterium*			*R. eutropha*		
	Cell mass (g/L)	PHA		Cell mass (g/L)	PHA	
		Yield (g/L)	%		Yield (g/L)	%
Glucose	2.45 ± 0.13	1.60 ± 0.09	65.3 ± 2.4	3.29 ± 0.13	2.01 ± 0.12	61.1 ± 2.3
Fructose	3.55 ± 0.15	2.18 ± 0.10	61.4 ± 2.3	3.94 ± 0.15	2.46 ± 0.14	62.4 ± 2.8
Whey	4.54 ± 0.18	2.93 ± 0.14	64.5 ± 2.7	6.05 ± 0.18	3.84 ± 0.17	63.5 ± 3.1

### Influence of process parameters on PHA production by *R. eutropha*

**Figure 1:**
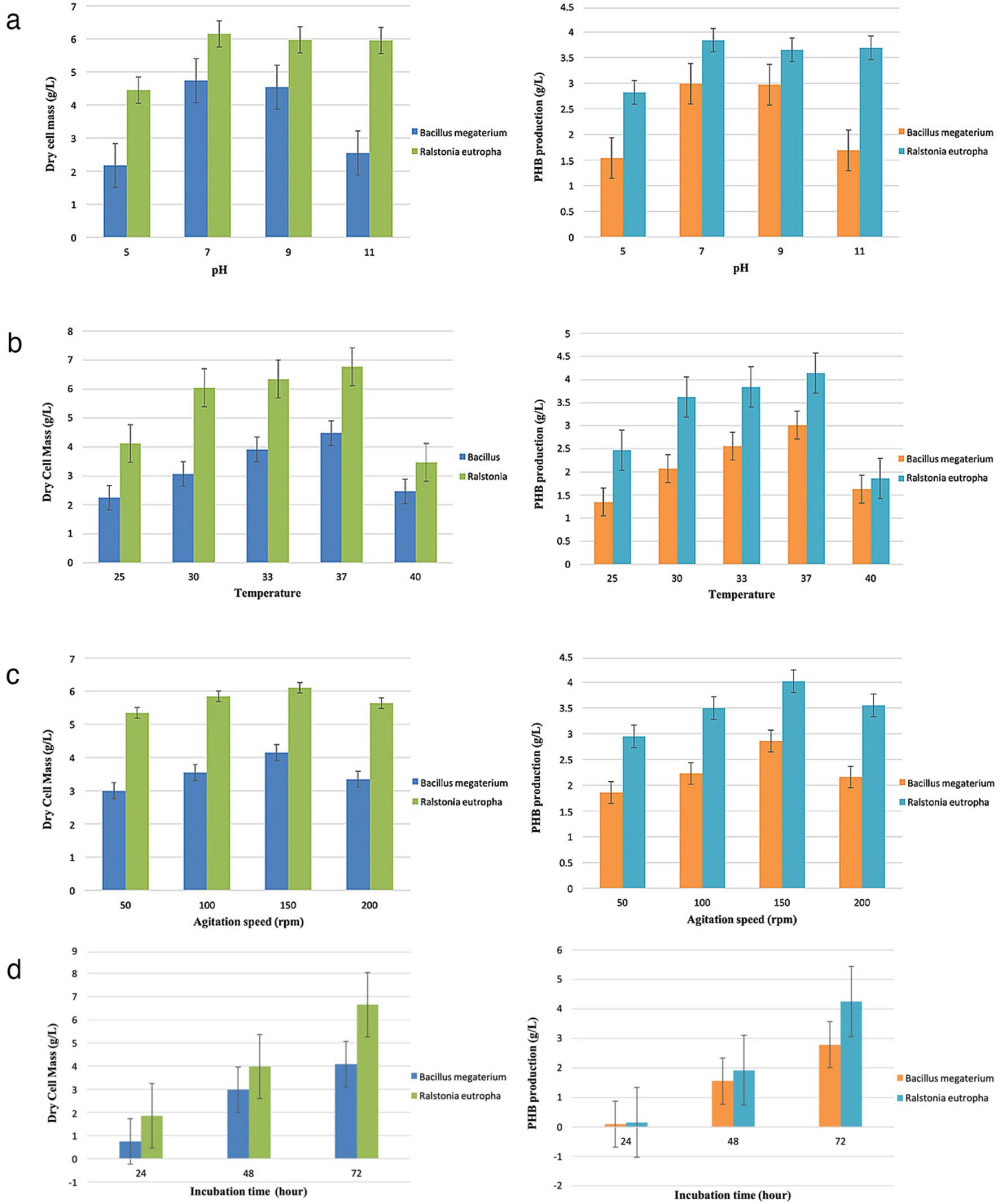
(**a**) Effect of different pH on dry cell mass and PHB production of two bacterial strains; (**b**) Effect of different temperatures on dry cell mass and PHB production of two bacterial strains; (**c**) Effect of different agitation speeds on dry cell mass and PHB production of two bacterial strains; (**d**) Comparison of Dry Cell Mass content and PHB production of *Bacillus megaterium* and *Ralstonia eutropha* as a function of incubation time.

To improve the PHA production by *R. eutropha* from whey hydrolysate, the physiological process parameters were evaluated at pH (5 – 11), temperature (25–40 °C), agitation rate (50–200 rpm), and incubation period (24–72 h) (Fig. 1). The optimum pH of 7 was noted for the high cell biomass (6.05 g/L) and PHA (3.84 g/L) (Fig. 1a). Under an acidic pH of 5, a significant decline in cell biomass to 4.45 g/L and PHA yield to 2.85% was noted by *R. eutropha*. In contrast, higher alkaline pH of up to 11 exhibited consistent cell biomass and PHA yields. These findings revealed that PHA accumulation by *R. eutropha* is favorable under neutral and higher alkaline conditions. Previously, a lower PHA production was recorded of 0.84 ± 0.14 g/L by *Pichia* sp. TSLS24 using Zobell marine agar medium (ZMA) supplemented with 2 gL^−1^ of sucrose to enrich the growth of yeast under alkaline pH 9^25^. The temperature has a notable influence on PHA production that can be associated with the survival strategy adopted by organisms over bioconversion at declined/elevated temperatures to the optimum conditions^26^. The optimum temperature of 37 °C was observed for efficient cell biomass and PHA production of 6.05, and 3.84 g/L, respectively (Fig. 1b). At 25 °C, a significant decline in cell biomass and PHA yield up to ~ 33%. In contrast, the lowest PHA yield and contents of 1.86 g/L and 53.6% were recorded at a higher incubation temperature of 40 °C, respectively. The optimum agitation rate of 150 rpm was noted for biomass and high PHA accumulation (Fig. 1c). The cell biomass *R. eutropha* increased with an increase in the incubation period from 1.86 g/L at 24 h to 6.65 g/L at 72 h (Fig. 1d). The optimum incubation of 72 h was noted for high PHA yield and contents of 3.84 g/L and 63.9%, respectively. At a higher incubation of 96 h, the partial decline in PHA contents to 57.8% can be associated with the diversion of accumulated PHA to depolymerization for survival benefits for *R. eutropha*^27^.

### Effect of amino acids supplementation on PHA Production by *R. eutropha*

**Figure 2:**
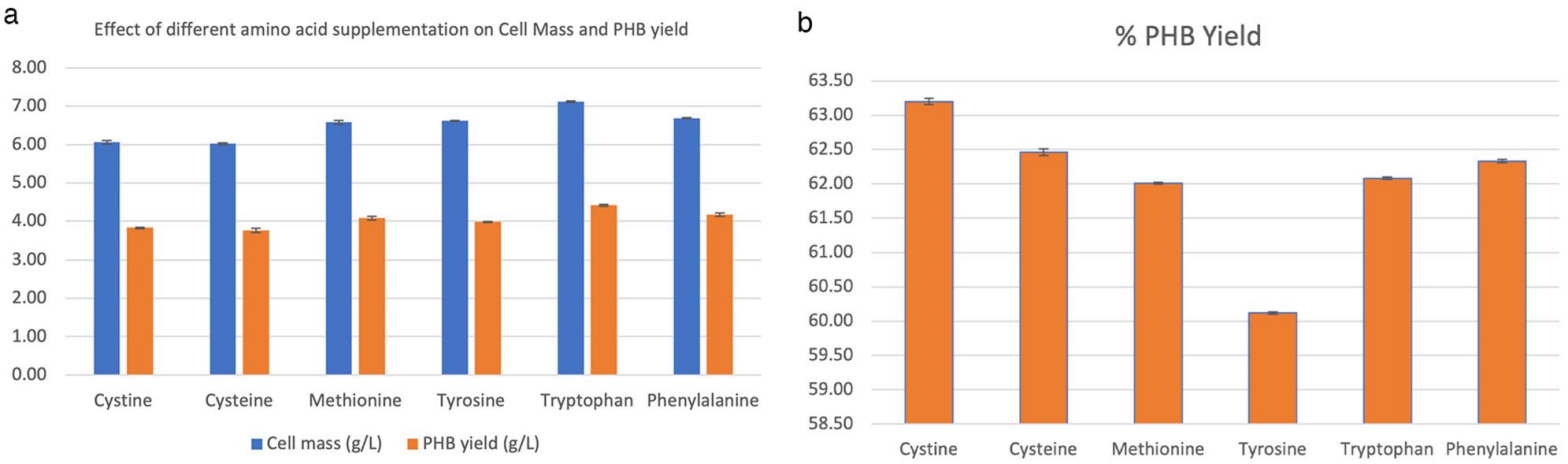
(**a**) Effect of amino acid supplementation on Cell Mass and PHB Yield; (**b**) PHB yield on supplementation with different amino acids expressed as a percentage.

Amino acids play a crucial role in cell biomass synthesis via their involvement in proteins manufacture. The bulk of chemical reactions that take place in cells are catalyzed by proteins. Several of the structural components of a cell are supplied by them^28^. Therefore, six amino acids (1 mg/mL) were supplemented to medium, including cystine, cysteine, methionine and tyrosine, tryptophan, and phenylalanine to evaluate their influence on the PHA contents and composition. On the supplementation of these amino acids, *R. eutropha* accumulated PHA in the ranges of 60.2–63.2% (Fig. 2). Here, PHA yields varied from 3.76–4.42 g/L. Among these amino acids, tryptophane supplementation to whey hydrolysate proved more beneficial for *R. eutropha* for accumulating high PHA content and yield of 62.1% and 4.42 g/L, respectively. Previously, supplementation of known amino acids (50 mg/L) in glycerol medium resulted in much lower PHB contents in the ranges of 8.86–26.2% by recombinant *E. coli*^29^. Further, by increasing amino acid contraction to 150 mg/L, cysteine was found suitable to enhance PHA yield up to 30.8% by *E. coli*. On the other hand, methionine, and isoleucine negatively influenced PHA with a remarkable decline in E. coli contents to 19.1 and 16.6% under similar conditions, respectively^29^. In contrast, *R. eutropha* in this study showed much better PHA contents up to 62.5% and 62% in the presence of cysteine and methionine, respectively (Fig. 2). The enhancement in PHA content by *R. eutropha* on supplementation of tryptophan might be associated with the fact that tryptophan production consumes more ATP than the biosynthesis of other amino acids, which requires less ATP^8^. When whey is supplemented with acetic acid (16%), butyric acid (26%), and lactic acid (58%), bacteria synthesized PHB only, while whey with valeric acid (4%), lactic acid (6%), butyric acid (13%), propionic acid (19%), and acetic acid (58%) bacteria have synthesized a copolymer made of (40%) PHV along with PHB (60%)^30^. *Haloferax mediterranei*, for example, uses whey to yield 66% CDW PHA (0.11 g L^−1^ h^−1^). High PHA producers, such as *Ralstonia eutropha* can accumulate PHAs up to 80% of its dry cell weight when grown on glucose^31^ & can make SCL-PHA and PHA biopolymers made up of 3HB, 3HV, and 4HB subunits^32^.

### Design of experiments and model fitting for PHA accumulation from whey hydrolysate supplemented with various concentrations of Tween 80 and tryptophan

The effective operating conditions for improving PHA content were examined using the response surface approach for *R. eutropha* from whey hydrolysate. Two process variables as, tryptophan and tween 80 concentrations, were selected at various levels, and 13 sets of experiments were then created using the central composite rotatable design (CCRD). The outcomes of the experimental trials are represented in Table 1. The cell biomass and PHA varied in the ranges of 5.41–6.81, and 3.56–4.90 g/L, respectively. The maximum PHA accumulation recorded of 4.90 g/L with yields of 77.4% by *R. eutropha* at concentrations of 37.5% for tryptophan, and 6.6% for the tween-80.

**Figure 3:**
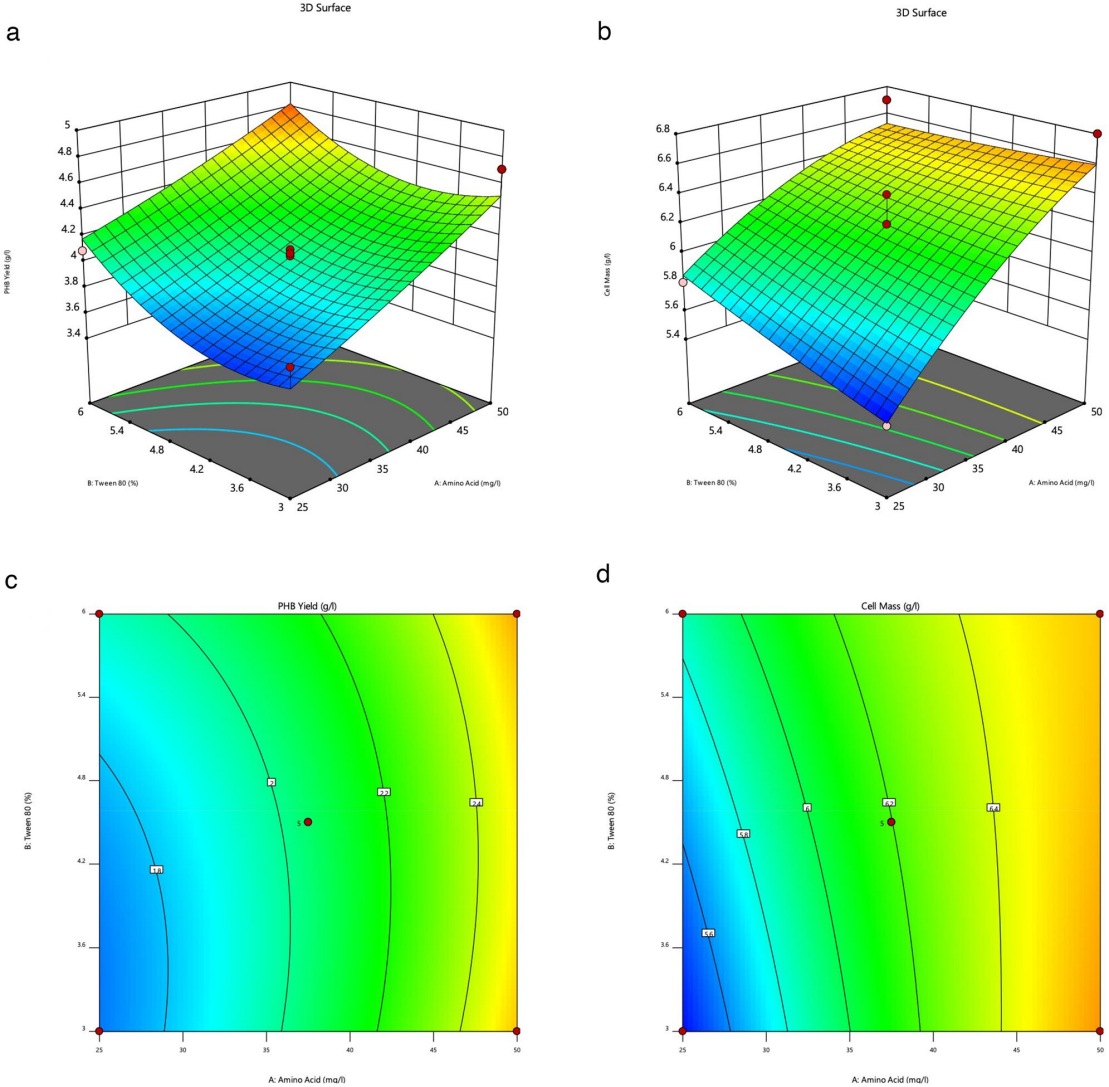
(**a**) 3D surface plot showing the relationship between amino acid and tween 80 concentration on the PHB yield (gL^−1^) in *Ralstonia eutropha* in the optimized sample; (**b**) 3D surface plot showing the relationship between amino acid and tween 80 concentration on the cell mass (gL^−1^) of *Ralstonia eutropha* in the optimized sample; (**c**) Contour plot showing the interactive effect of amino acid and tween-80 concentration on PHB content (gL^−1^) in *Ralstonia eutropha* in the optimized sample; (**d**) Contour plot showing the interactive effect of amino acid and tween-80 concentration on cell mass (gL^−1^) of *Ralstonia eutropha* in the optimized sample.

Tryptophan and tween-80 showed a beneficial effect on PHA accumulation, as seen in Fig. 3a. The 3D surface and contour plot clearly evidenced that increasing the concentrations of tween-80 up to 6.6% v/v and tryptophan up to 37.5% improved PHA accumulation by *R. eutropha* (Figs. 3a-c). The biomass of *R. eutropha* was raised by the amino acid concentration. In contrast, the biomass was adversely lowered by the increase in the tween-80 concentration, as indicated in the 3D plot's boundary (Fig. 3b). Cell biomass was found to be at its highest value of 6.81 g/L at 50 mg/L of tryptophan and 3% of tween-80, according to a contour plot illustrating the interaction impact of these two concentrations (Fig. 3c). A lower PHA accumulation can be correlated by a substantial portion of acetyl-CoA alteration to metabolic pathways that compete with synthesis of PHA, such as the formation of acetate, fatty acids, and amino acids^8^. An increasing concentration of up to 50 mg/L of tryptophan raised cell biomass. In contrast, a significant decline in biomass was noted on increasing tween-80 concentration up to 6% (Fig. 3d). However, there was no synergistic effect on PHA accumulation noted as the concentration of tryptophan increased. This might be explained by the PHA synthase enzyme being inhibited by its substrate^8^.

### Up-scaling of PHA production

**Figure 4:**
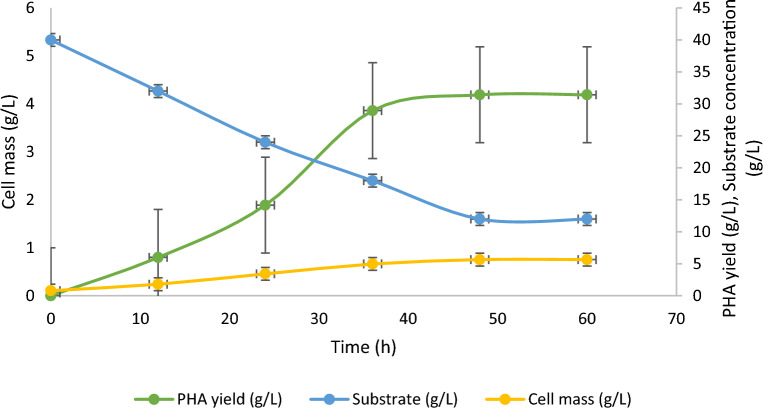
Kinetics of PHA production under optimized condition in 7.5 L bioreactor (working volume: 3.0 L).

The up-scaled production profile of PHA production by *R. eutropha* from whey hydrolysate is presented in Fig. 4. At the log phase of growth of 12 h, the biomass growth increased to 5.65 g/L at 48 h of incubation. At 3-L of culturing, the maximum PHA content and yields were noted 4.19 g/L, and 74.2%, respectively. During PHA accumulation, the substrate consumption was observed at 79.0% from the initial feed of 40 g/L. The kinetic measurements reveal that PHA yield (YP/x) in terms of cell biomass was recorded at 0.72 with a productivity of 0.19 g/L/h. Previously, 0.024 g/L/h PHA productivity was observed in *Bacillus* sp. from media containing glucose, yeast extract, peptone and a few inorganic salts^33^ which is lower than the present finding. Similarly, 0.071 g/L/h PHA productivity in *Pseudomonas chlororaphis* grown on animal derived waste^34^.

### Characterization of produced PHA by *R. eutropha*

PHA granule formations occurred in cells as PHA oligomers create a micelle-like form or plasma membrane buddings off, leaving a granule coated in a monolayer of lipids^35^. The visualization of *R. eutropha* TEM micrographs depicts rod-shaped cells containing white inclusions of PHA granules. Overall, PHA granules are generated both at the cytoplasmic membrane and dispersed evenly throughout the cytoplasm, which is consistent with the previous report on PHA granules formation by *Bacillus cereus* from pea-shells hydrolysate^18^.

**Figure 5:**
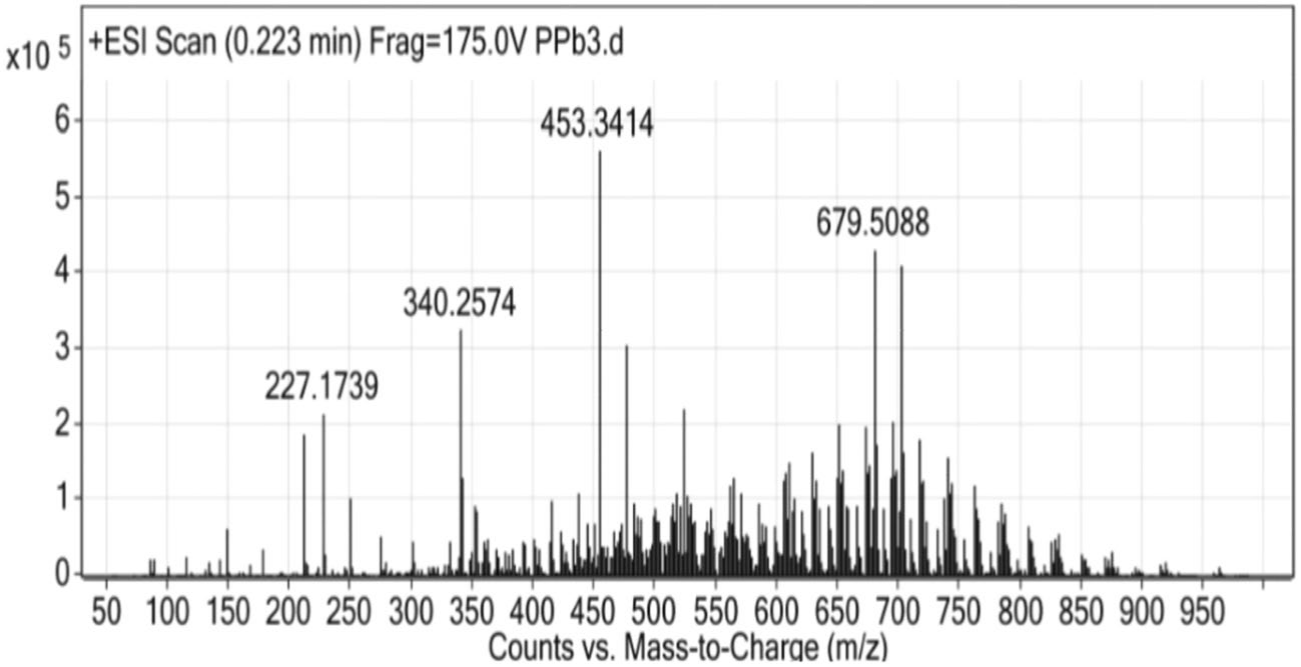
ESI–MS of the fraction of PHA polymers formed after the partial thermal degradation of the PHA synthesized by Ralstonia eutropha in optimized conditions where tryptophan and tween 80 are supplemented to whey medium.

The controlled PHA chemical decomposition can occur in several ways via acetic acid salts that generate oligomers with the same composition and sequence distribution of monomer units^36^. To validate the existence of P(3-HB) monomers, ESI/MS analysis was performed to determine the chemical structure of PHA produced by *R. eutropha* from whey hydrolysate. The ESI–MS spectrum of oligomers produced by PHA breakdown in the presence of potassium acetate is shown in Fig. 5. The peak-to-peak mass increment in the ESI–MS spectrum was noted of 86 Da, which is equivalent to the mass of the 3-hydroxybutyrate (3-HB) repeating unit. Other series of ions recorded correspond to the sodium adduct of poly (3-HB) with crotonate and carboxyl end groups. The molar mass of the 3-hydroxyhexanoate (HH, 114 Da), and 3-hydroxyvalerate (HV, 100 Da) co-monomeric units differed by 14 Da across surrounding signals (3-HB, 86 Da) within the clusters. The ESI mass spectrum of PHA polymers (HB, HV, and/or HH subunits) revealed a dispersion of singly charged sodium adducts of the distinct PHA polymer chains (terminating with unsaturated and carboxylic end groups) due to their strong sensitivity for alkali metals (particularly sodium). The present investigation showed PHA copolymers i.e., HB-HH, HB-HV in whey supplemented media which is in correlation with previous findings of Kowalzuck et al., who reported HB-HV oligomers in oxidized polyethylene wax supplemented media using *R. eutropha*^37^. The composition of the PHAs obtained from *C. testosteroni* during growth on variety of vegetable oils showed 3-hydroxyoctanoic acid and/or 3-hydroxydecanoic acid^38^.

**Figure 6:**
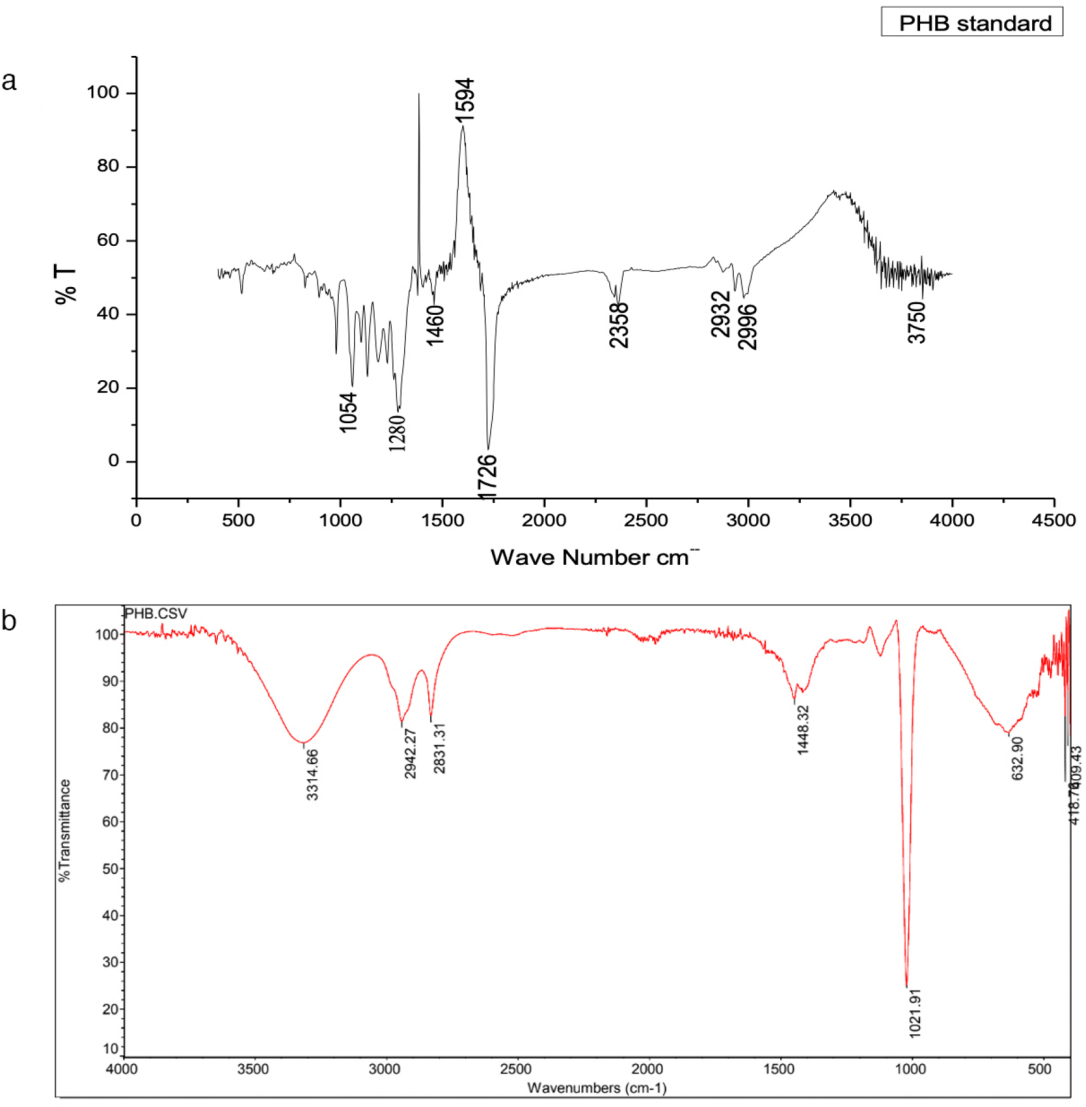
FTIR spectra of PHA copolymers synthesized by *Ralstonia eutropha* from whey.

The characterization of produced PHA was validated by FTIR spectra (Fig. 6). The peaks at 1021 cm^−1^ represent the ester bond. The stretching of the C–O and -CH bond present in the ester group was noted at peaks ~ 1700 and ~ 1300 cm^−1^, respectively^39^. The peak at 1377 cm^−1^ indicates the occurrence of a symmetric bending of the -CH_3_ group. Further, the asymmetric bending of –CH_2_ and –CH groups correlated to the peaks at 1448 and 2942 cm^−1^, respectively. The peak at 3314 cm^−1^ correlated to a terminal –OH group^8^.

## Conclusion

In this study, PHA-producers *B. megaterium* and *R. eutropha* feasibility to produce PHAs from spilled whey as low-cost raw materials feed was demonstrated. Initial screening suggested that *R. eutropha* can efficiently produce PHAs up to twofold higher compared to *B. megaterium*. The supplementation of amino acids and tween 80 in feed showed remarkable enhancement in PHAs production ~ 50-fold under optimized conditions in medium optimization over uses of whey alone as a feed.

### Supplementary Information

Supplementary Information.

## Data Availability

All data generated or analyzed during this study are included in this published article.
