# Author Correction: Production, optimization, scale up and characterization of polyhydoxyalkanoates copolymers utilizing dairy processing waste

**DOI:** 10.1038/s41598-024-61698-9

**Published:** 2024-05-13

**Authors:** Tejaswini Dhanaji Patil, Saptaneel Ghosh, Aparna Agarwal, Sanjay Kumar Singh Patel, Abhishek Dutt Tripathi, Dipendra Kumar Mahato, Pradeep Kumar, Petr Slama, Ales Pavlik, Shafiul Haque

**Affiliations:** 1 Department of Dairy Science and Food Technology, Institute of Agricultural Sciences, Banaras Hindu University, Varanasi, Uttar Pradesh 221005 India; 2 Department of Food and Nutrition Science, Lady Irwin College, Delhi University, New Delhi, 110001 India; 3 Department of Chemical Engineering, Konkuk University, Seoul, 05029 Republic of Korea; 4 School of Exercise and Nutrition Sciences, CASS Food Research Centre, Deakin University, Burwood, VIC 3125 Australia; 5 Department of Botany, University of Lucknow, Lucknow, 226007 India; 6 Department of Animal Morphology, Physiology and Genetics, Faculty of AgriSciences, Mendel University in Brno, 61300 Brno, Czech Republic; 7 Research and Scientific Studies Unit, College of Nursing and Health Sciences, Jazan University, 45142 Jazan, Saudi Arabia; 8 Gilbert and Rose‑Marie Chagoury School of Medicine, Lebanese American University, Beirut, 1102 2801 Lebanon; 9 Centre of Medical and Bio‑Allied Health Sciences Research, Ajman University, 13306 Ajman, United Arab Emirates

Correction to: *Scientific Reports* 10.1038/s41598-024-52098-0, published online 18 January 2024

The Author Contributions section in this Article was incomplete.

 “T.D.P., A.D.T., P.S., A.P., S.H. and S.K.S.P. conceptualized the experiment. T.D.P., A.D.T. and A.A did the methodology and validation. T.D.P., A.D.T. conducted the formal analysis and investigation. T.D.P., A.D.T., and S.K.S.P. wrote and prepared the original draft. A.D.T., P.S., A.P., S.H., and S.K.S.P. conducted the writing, review and editing. A.A. helped in visualization. A.D.T. and A.A. supervised the experiment. S.H. and D.K.M. edited the manuscript. T.D.P., A.D.T. and A.A. performed analytical work. All authors have read and agreed to the published version of the manuscript.”

now reads,

“T.D.P., A.D.T., P.S., A.P., S.H. and S.K.S.P. conceptualized the experiment. T.D.P., A.D.T. and A.A. did the methodology and validation. T.D.P., A.D.T. conducted the formal analysis and investigation. T.D.P., A.D.T., and S.K.S.P. wrote and prepared the original draft. A.D.T., P.S., A.P., S.H., and S.K.S.P. conducted the writing, review, and editing. A.A. helped in visualization. A.D.T. and A.A. supervised the experiment. S.H. and D.K.M. edited the manuscript. T.D.P., A.D.T., P.K., and A.A. performed analytical work. All authors have read and agreed to the published version of the manuscript.”

The original Article has been corrected.

