# Supplementary figures and images for: Production, optimization, scale up and characterization of polyhydoxyalkanoates copolymers utilizing dairy processing waste
Source: Sci Rep. 2024 Jan 18;14:1620. doi: 10.1038/s41598-024-52098-0 (PMC10796949; doi:10.1038/s41598-024-52098-0)

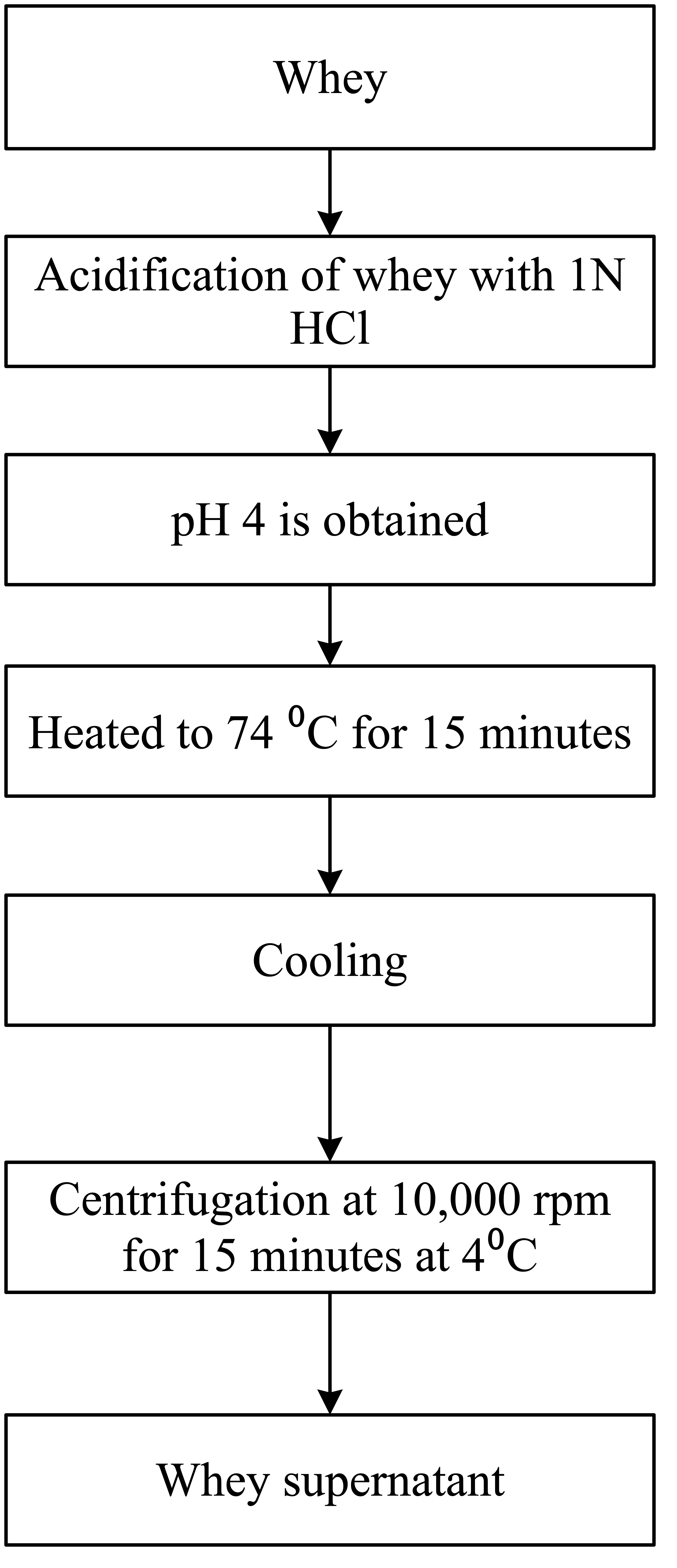


**Supplementary Fig S1**: Flow chart of whey pre-treatment

Supplement: Supplementary file 1 — Supplementary Information. [file 41598_2024_52098_MOESM1_ESM.docx]
